# Reuse of lead glass sludge in the fabrication of thermally insulating foamed glass with outstanding properties and high Pb-stabilization

**DOI:** 10.1007/s11356-022-19184-0

**Published:** 2022-02-18

**Authors:** Hamdy A. Abdel-Gawwad, Mona S. Mohammed, Mohammed A. Arif, Hamada Shoukry

**Affiliations:** 1 Raw Building Materials and Processing Technology Research Institute, Housing and Building National Research Center (HBRC), Cairo, Egypt; 2 Department of Chemical Engineering and Pilot Plant, National Research Centre, Cairo, Egypt; 3 Department of Chemistry, Faculty of Science, Zagazig University, Zagazig, Egypt; 4 Building Physics Institute (BPI), Building National Research Center (HBRC), Housing &, Cairo, Egypt

**Keywords:** Foamed glass, Sintering, Leaching; Porosity, Thermal conductivity, Compressive strength

## Abstract

This study represents the sustainable/safe consumption of lead glass sludge (LGS) in the fabrication of thermally insulating foamed glass via sintering (750–950º C) and chlorination processes. The impact of selected additives including calcium chloride (CaCl_2_) and sodium hydroxide (NaOH) on the foaming efficiency and Pb-stabilization has been deeply investigated. LGS is mainly lead silicate material with considerable content of calcium carbonate, which acts as foaming agent during sintering process. The newly developed foamed-materials exhibited thermal conductivity of 0.054–0.136 W/m.K, density of 0.23–1.10 g/cm^3^, porosity of 63.3–92.6%, and compressive strength of 0.10–2.69 MPa. X-ray diffraction proved that the immobilization mechanism was attributed to the transformation of free Pb within LGS into insoluble ganomalite Pb_9_Ca_5_MnSi_9_O_33_ phase. Adding NaOH enhanced the foaming process accompanied by a significant reduction in Pb-leaching. Incorporating CaCl_2_ has resulted in a retardation in Pb-leaching, which associated with Pb-stabilization and Pb-vaporization. In an attempt to reduce CO_2_-emission, the potential use of alkali-rich-wastewater (AW) as eco-friendly alkali source in lieu of NaOH was studied. Regardless of the variation in Pb-concentrations in leachates, all samples recorded Pb-concentrations lower than the safe limit (≤ 5 mg/l), achieving Pb-immobilization of 95.98–99.87%. The significantly reduced thermal conductivity and enhanced Pb-immobilization efficiency along with the reasonable compressive strength summarize the major innovation presented in this study.

## Introduction

Lead (Pb)-bearing-wastes are categorized as accumulative hazardous pollutants, which cause serious problems to the human health and environment (WHO, 2019). The united nation environment program showed that there is no safe limit for exposure to Pb, as the lowest dose causes problems in the human nervous system (UNEP 2013). Stabilization/solidification of Pb-contaminated-wastes is mandatory to overcome the hazardous effect of this metal. Accordingly, different research approaches were implemented to transform the hazardous lead into safe and inactive materials. The immobilization of Pb using Portland cement (Wang et al. 2018; Niu et al. 2018), alkali-activated cement (El-eswed 2020; Li et al. 2021), and reactive magnesia (Shen et al. 2018, 2019a, b; Abdel-Gawwad et al. 2021) has been extensively addressed. Physical immobilization of Pb within calcium silicate hydrate phase (Wang et al. 2018), the incorporation of Pb within aluminosilicate network (El-eswed et al. 2017), and the formation of Pb hydroxide, cerussite, and hydrocerussite (Zhan et al. 2020) are the main suggested mechanism of Pb-stabilization via Portland cement, alkali-activated cement, and reactive magnesia, respectively.

Thermal treatment of Pb-bearing-wastes is one of the most effective methods for stabilization and removal of Pb. Adding chloride salt before thermal treatment greatly enhanced the removal of Pb via chlorination/evaporation process (Yu et al. 2013; Nowak et al. 2010; Nowak et al. 2012). Chloride can chlorinate PbO at 800–900 ºC to yield PbCl_2_ with lower melting point compared with PbO_,_ resulting in an enhancement in Pb volatilization. The presence of mineral matter (Si and Al) can hinder Pb-evaporation, which associated with the formation nonvolatile Pb-metallic species (Yu et al. 2013). Magnesium chloride, calcium chloride, and sodium chloride salts are the main chloride salts used in the chlorination of Pb (Nowak et al. 2012). Poly vinyl chloride (PVC) was previously used as organic chloride source. Nowak et al. 2010, 2012) found that the chlorination/evaporation of Pb via calcium chloride is more efficient than sodium chloride. Calcium oxide was found to exhibit a retardation effect on PVC-induced Pb-volatilization; whereas it has no effect on the direct chlorination of sodium chloride-containing-system (Wang et al. 2017).

The environmental pollution resulted from glass manufacturing can be partitioned into three types: solid waste, waste water, and air emission (FC 2007). Regarding lead glass industry lead component was used as the main ingredient in this manufacturing. Almost 30% of lead used escapes during manufacturing stages including transportation and batch preparation, melting process in the furnace, and grinding and polishing (Pechnikov et al. 1996). Lead glass sludge (LGS) is categorized as a hazardous waste resulted during the grinding and polishing of lead glass. Lead glass industry annually produces 6.3 million tonnes all over the world (Bursi et al. 2017). Approximately 20 tonnes/day was resulted from lead glass manufacturing in Egypt (Elkersh 2014). LGS has been transformed into safe building material via its incorporation in alkali-activated slag (Abdel-Gawwad et al. 2019; 2020a) and magnesia-based bricks (Abdel-Gawwad et al. 2021).

Apart from the thermal treatment/chlorination of Pb-containing-wastes demonstrate high efficiency in Pb-removal, the rest ash from incineration of solid wastes contributes to environmental problems including falling landfill space and landfill contamination. Accordingly, the application of thermal treatment and chlorination for the remediation of Pb-bearing-materials through the production of a widely used and a safe product is the main challenge. Therefore, the novel contribution of the present work is to utilize chlorination and thermal treatment for immobilization/removal of Pb-containing-glass sludge through the fabrication of thermally insulating glass foam with high stability to acidic media. The impact of calcium chloride (chlorination) and sodium hydroxide addition on the foaming process and Pb-remediation within sintered-LGS. Owing to the manufacture of sodium hydroxide generates high CO_2_ emission (Turner and Collins 2013; McLellan et al. 2011), alkali-rich-wastewater (AW) from aluminum pots manufacturing was used as eco-friendly alkali source. The mechanism of Pb-stabilization through thermochemical treatment of LGS has been also addressed.

## Experimental

### Materials

As the main starting material of foamed-glass, LGS (which was resulted from polishing of glass crystal) was supplied from ASFOUR Company for Glass Crystals Industry (Cairo, Egypt). Ultra-pure sodium hydroxide (NaOH) and calcium chloride (CaCl_2_), as additives for LGS, were purchased from Fisher Scientific Chemical Company (UK). AW from aluminum pots manufacturing, was used as an alternative to chemical NaOH. AW exhibits Na^+^ content of 64,345 mg / l and pH of 13.15 ± 0.2.

### Preparation of foamed-LGS

The formation of foamed-LGS includes three main stages, i.e., LGS-grinding (grinding was conducted in a ball mill to achieve fineness of 6200 cm^2^/g), wet mixing and molding, and sintering at elevated temperatures. Simply, the LGS powder was mixed with water at water to LGS weight ratio of 0.40. The workable paste was transferred into stainless steel mold of 50 × 125 × 25 mm (width × length × height, respectively), followed by sintering at different elevated temperatures. Owing to high-volume change occurs after sintering process, the workable LGS-paste should spread along the surface’s mold to yield a layer with an average height of 3 mm to avoid get off the foamed-LGS sample from the mold after sintering. The foamed materials left to cool in an oven for 24 h, then carefully demolded. As shown in Table 1, thirteen mixtures have been prepared in the present work. Four mixtures were prepared by the exposure of LGS mixed with tap water (TW) to 750, 800, 850, and 950 ºC for holding time (sintering time) of 2 h. At constant mixing water (TW) and sintering temperature (850 ºC), two mixtures have been prepared at different holding times of 1 and 3 h. Other three mixtures, in which LGS was individually mixed 1, 3, and 5 wt.% NaOH, were synthesized at sintering temperature of 850 ºC and holding time of 2 h. CaCl_2_ solutions with concentrations of 1, 3, and 5 wt.% were mixed with LGS, then exposed to the same sintering conditions to yield three foamed-LGS samples. The last foamed sample was synthesized by sintering LGS mixed CaCl_2_ solution with concentration of 3 wt.% using AW as a solvent. Three samples of each mixtures were prepared and characterized using different experimental methods (which were discussed below).

**Table 1:** Details of mixing proportions and sintering conditions of foamed LGS

Mix notation	LGS	NaOH	CaCl_2_	Temperature	Holding time	Type of mixing water
	Wt.%			ºC	hour	
F-LGS-750 (2 h)	100	-	-	750	2	TW
F-LGS-800 (2 h)	100	-	-	800	2	TW
F-LGS-850 (1 h)	100	-	-	850	1	TW
F-LGS-850 (2 h)	100	-	-	850	2	TW
F-LGS-850 (3 h)	100	-	-	850	3	TW
F-LGS-950 (2 h)	100	-	-	950	2	TW
F-LGS-850 (2 h)-SH1	100	1	-	850	2	TW
F-LGS-850 (2 h)-SH3	100	3	-	850	2	TW
F-LGS-850 (2 h)-SH5	100	5	-	850	2	TW
F-LGS-850 (2 h)-CC1	100	-	1	850	2	TW
F-LGS-850 (2 h)-CC3	100	-	3	850	2	TW
F-LGS-850 (2 h)-CC5	100	-	5	850	2	TW
F-LGS-850 (2 h)-CC3 / AW	100	-	3	850	2	AW

### Methods

The effectiveness of foaming process at different conditions was evaluated by conducting different experimental methods on the produced foamed materials. Volume expansion% (V_exp_: after sintering process) was determined according to the following equation:

$$\mathrm{Vexp }\left(\mathrm{\%}\right)=\frac{Vi-Vs}{\mathrm{Vi}}\times 100$$

*Vi* and *Vs* are the initial and final volumes after sintering process, respectively.

The bulk density (g / cm^3^) was simply carried out according to ASTM C303 (2021) by dividing dry mass to volume ratio of the sintered sample. He-pycnometer (Micromeritics AccuPyc 1330, Norcross, GA) was used to determine the amounts of total, open, and closed porosity. Before measuring compressive strength, the foamed materials were cut into 50 × 50 × 50 mm (width × length × height, respectively). Followed ASTM C109M (2020), compressive strength was conducted on three samples of each mixture. This test was operated by means of five tons of German-Bruf-Pressing Machine with a maximum capacity of 175 kN.

The leaching of Pb from LGS before and after sintering process was measured by acetic acid leaching test. According to toxicity characteristic leaching procedures (TCLP: USEPA 1992), 5 g of sample was suspended in 100 ml of 0.1 M acetic acid solution with pH of 2.9 ± 0.05. Thereafter, shaking was applied on the acetic acid solution containing sample for 18 h, followed by filtration using Whitman filter paper (Grade 41). The total bioavailable Pb within LGS was determined by mixing 0.25 g of a powdered-sample with 9 mL of HNO_3_ with a concentration of 70 wt.% and 3 mL of HCl with a concentration of 36 wt. %. The sequential Pb leaching test is categorized as an important method to determine the behavior of Pb-leaching (Shen et al. 2018, 2019a, b). The Pb leaching was divided into four fractions including exchangeable, acid soluble, organic bound, and non-bioavailable Pb-fractions. Exchangeable Pb-fraction was determined by mixing 1 g of sintered and un-sintered LGS with 8 mL of MgCl_2_ solution (0.5 M) at pH of 7 (NaOH was used to adjust pH). The suspended solution was shaken at 250 rpm and a temperature of 23 ± 2 °C for 20 min, followed by filtration. Before conducting acidic soluble Pb-fraction, the solid residue from the exchangeable Pb-fraction step was washed many times by distilled water to remove any contaminants. The acidic soluble fraction test was carried out by suspending the washed solid residue from the previous test in 8 mL of 1 M sodium acetate solution at pH 5 (pH was adjusted by 0.1 M acetic acid), followed by shaking at 250 rpm and a temperature of 23 ± 2 °C for 5 h. To determine the Pb bound to organic matter, the washed solid residue from acidic soluble Pb-fraction test was shaken in 20 mL of 30% H_2_O_2_ and 0.02 M NH_2_OH at pH of 2.0. This mixture was mixed with 20% HNO_3_ and 3 mol ammonium acetate. Finally, the non-bioavailable Pb-fraction was estimated by subtracting the total bioavailable Pb (7953 mg/Kg) from four Pb-fractions.

After each Pb-fraction, centrifuging and filtration were applied to separate leachate from the solid residue. The insoluble residue was washed by 20 mL of deionized water to remove any impurities on its surface, before its usage in the next step. The filtrate (leachate) from each Pb-fraction was acidified and diluted prior to Pb measurement using inductively coupled plasma atomic emission (ICP-AE) spectroscopy instrument (ULTIMA2, Horiba, Japan). The sequential Pb-extraction test was conducted three times, and the average was calculated and recorded.

### Instrumental analyses

X-ray diffraction (XRD) was conducted to identify the phase composition of LGS and the change occurred after the sintering LGS sample at different conditions. XRD was conducted using a Philips PW3050/60 diffractometer. Sample was scanned under the following conditions: radiation source of Cu Kα at ʎ = 1.5419 Ǻ; divergence slit adjusted at 10 mm in-plane; scanning range of 5–50 2θ°; scanning rate of 0.5° 2θ min^−1^; sampling interval of 0.02° 2θ. X-ray florescence (XRF: Xios PW 1400) was used for a quantitative analysis to determine the oxides analysis of LGS. Thermogravimetric analysis (TGA) and its derivative (DTG) was applied to identify the weight loss related to different phases within LGS. Differential thermal analysis (DTA) also was used to determine the softening point of silicate within LGS. TGA and DTA was conducted using a DT-50 thermal analyzer (Schimadzu Co-Kyoto, Japan). These tests were performed by weighing 20 mg of a powdered-LGS IN Pt-crucible, followed by heating in N_2_-atmosphere at 1000 ºC with a heating rate of 10 ºC/minute. The pore system of the foamed LGS was investigated by Leica S8 APO stereo-microscopy.

The transient line heat source method for measuring thermal properties of porous solid structures has been effectively adopted in many previous studies (Kim et al. 2003; Demirboga 2003; Al-Jabri et al. 2021). In the current study, KD2 Pro [Meter group, Washington] thermal properties analyzer was used to determine the thermal conductivity of the developed composites according to ASTM D5334-14. The dual needle 1.3 mm dia. × 30 mm long, 6 mm spacing was used. Two parallel grooves were created in test specimens with the aid of a 6-mm spacer; thermal grease was used to avoid the contact thermal resistance of the probe. Three specimens were tested for every mix and the average values were reported. To eliminate the influence of moisture content on values of thermal conductivity, the test specimens were dried at 100 °C for 24 h before testing.

## Results

### Characterization of LGS

**Fig. 1:**
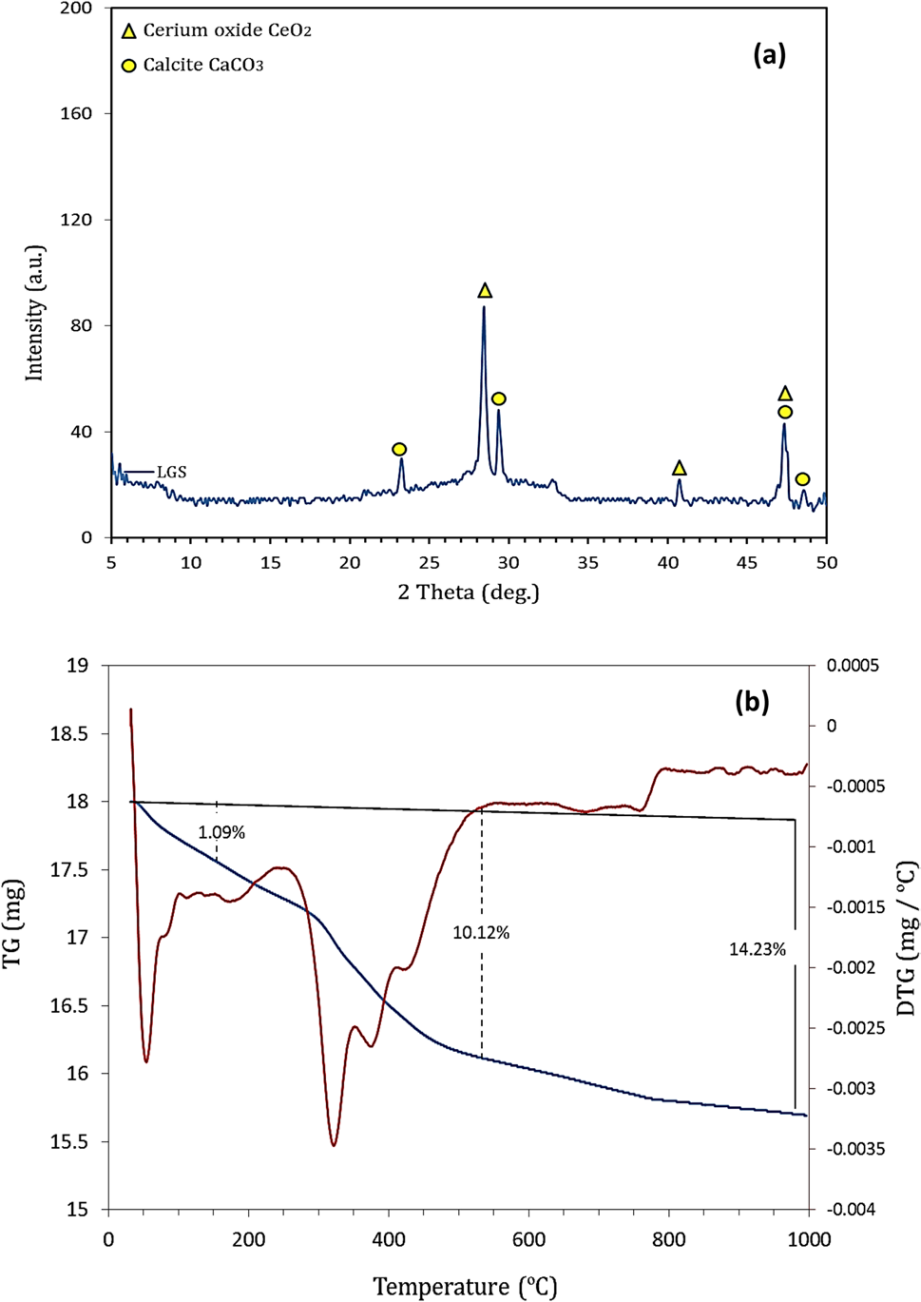
XRD-pattern (**a**) and TG/DTG-curves (**b**) of LGS

XRD shows (Fig. 1) that LGS exhibits amorphous pattern with the appearance of crystalline peaks affiliated to calcium carbonate (CaCO_3_) and silicon. SiO_2_ and PbO represent approximately 60% of the total oxides of LGS (Table 2), suggesting that LGS is lead silicate-rich-waste. Stoichiometrically, the CaCO_3_ content within LGS can be estimated by means of CaO content, as 3.69% CaO is resulted from the dissociation of 6.58% CaCO_3_. TG/DTG-curve (Fig. 1) proves that there are three weight losses affiliated to dehydration of free water (50–70 ºC), dissociation of organic matter (200–550 ºC), and the decomposition of CaCO_3_ (700–850 ºC). This indicates that the loss on ignition listed in Table 2 is the summation of weight losses of these phases. As identified by TG-curve, the weight loss affiliated to organic matter equals 10.12%, representing 71% of the total weight loss of LGS. On the other hand, the weight loss affiliated to CO_2_ (3.02%) is mainly resulted from the dissociation of 6.86% CaCO_3_. This value is nearly closed to that (6.58%) estimated from XRF-analysis (Table 2), suggesting the identified CaO content is mainly affiliated to CaCO_3_ within LGS.

**Table 2:** Oxides compositions of LGS

Notations	Oxides wt. %													
	SiO_2_	CaO	MgO	Fe_2_O_3_	Al_2_O_3_	SO_3_	Cl	Na_2_O	K_2_O	PbO	CeO_2_	MnO	La_2_O_3_	LOI*
LGS	41.20	3.69	0.288	8.61	1.23	0.44	0.07	1.10	3.85	18.65	4.72	0.32	1.78	14.03

^*^LOI loss in ignition was conducted at 950 ± 50 °C at 0.5 h soaking time according to ASTM C114

### Characterization of LGS foams

**Fig. 2:**
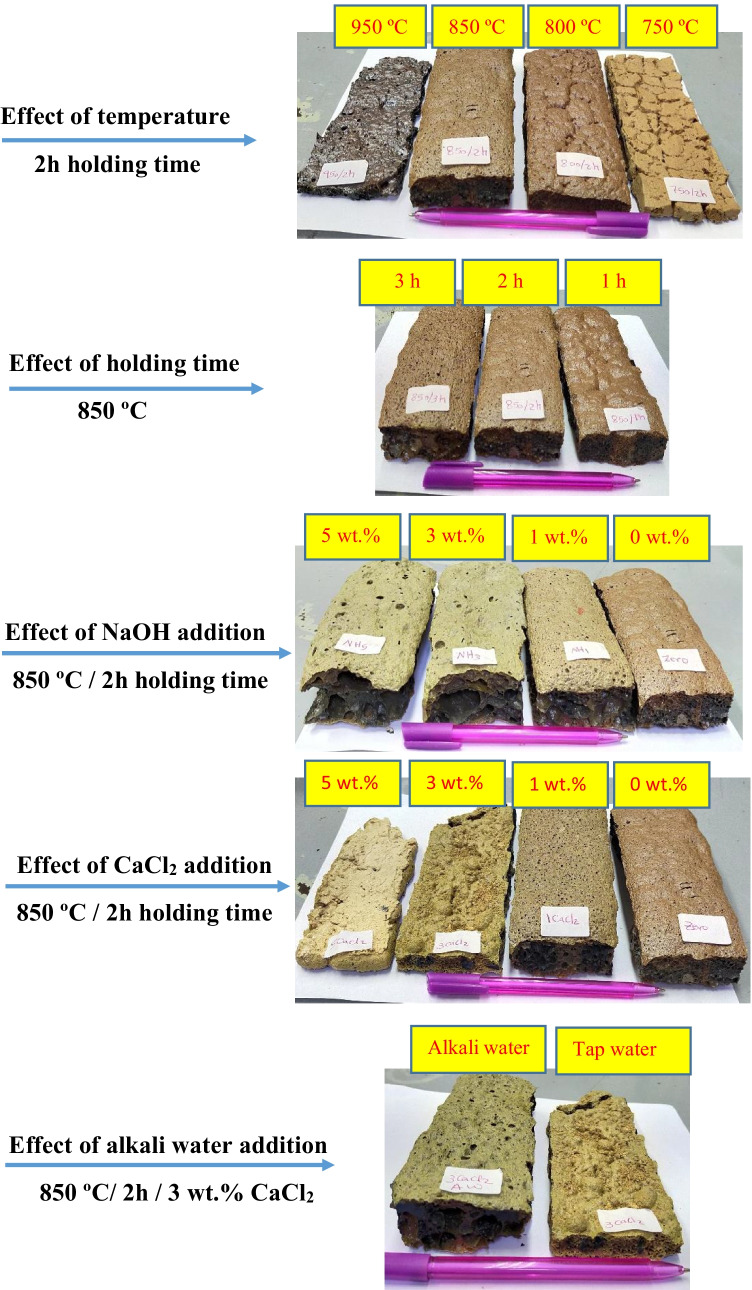
Digital photos of LGS foams synthesized at different conditions

The sintering LGS has resulted in the formation of foamed-materials with different pore systems depending on treatment conditions (Fig. 2). The dissociation of CaCO_3_ within LGS during thermal treatment and the softening amorphous silicate are the main reasons behind foaming process. CaCO_3_ is regarded as an internal foaming agent, as it decomposed to yield calcium oxide and carbon dioxide (CO_2_) gas, resulting in the formation of cellular materials (Chen et al. 2012; Praveen Kumar et al. 2015; Saparuddin et al. 2020).

**Fig. 3:**
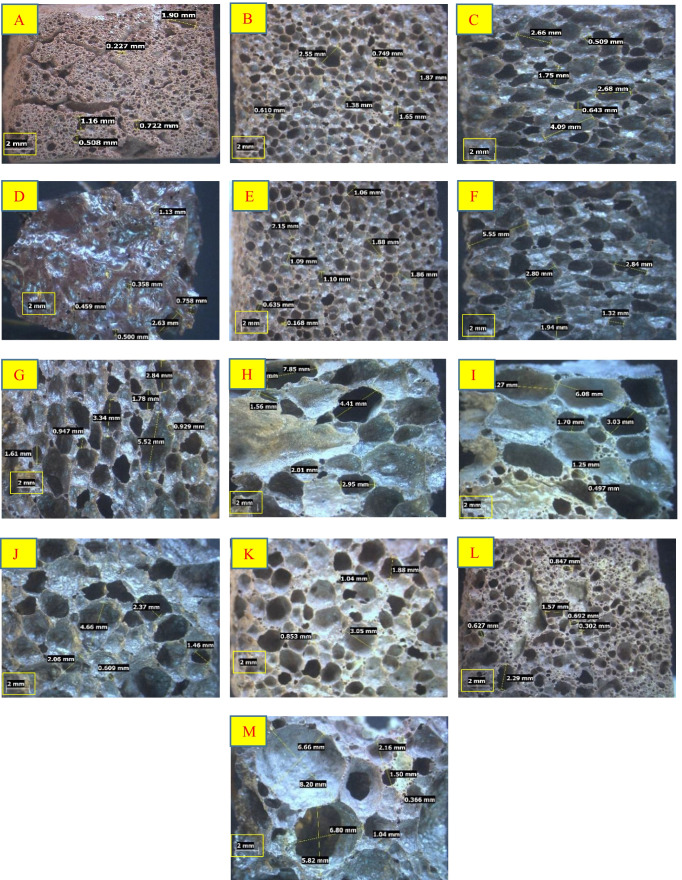
Stereomicroscopic photos of foamed LGS samples synthesized at 750–950 ºC for 2 h holding time (**A**–**D**), at 850 ºC for 1 and 3 h holding time (**E** and **F**, respectively), at 850 ºC for 2 h holding time in the presence of 1, 3, and 5 wt. % NaOH (**G**, **H**, and **I**, respectively), at 850 ºC for 2 h holding time in the presence of 1, 3, and 5 wt. % CaCl_2_ (**J**, **K**, and **L**, respectively), and at 850 ºC for 2 h holding time in the presence of 3, CaCl_2_ dissolved in AW (**M**)

The stereomicroscopic photos (Fig. 3) proved that the size, shape, and number of pores within foamed the materials strongly influenced by sintering temperature, holding time, NaOH and CaCl_2_ content, and type of mixing water. At constant holding time (2 h), the foamed material prepared at 750 ºC exhibits spherical- and elongated-shaped pores with small diameter ranged from 0.22 to 1.90 mm. Increasing sintering temperature up to 800 ºC causes the formation of foamed-LGS containg closed and opened spherical cells with diameter of 0.26–2.55 mm. A significant increase in pore diameter (0.5–4.09 mm) was detected in the case of foamed material synthesized at 850 ºC and holding time of 2 h. The exposure of LGS to 950 ºC results in structural densification where the lattice structure collapses to a solid with reduced pore size (0.35–1.13 mm).

At constant sintering temperature (850 ºC), increasing holding times (1–3 h) leads to an increase in pore volume. Additionally, the thermochemical treatment of LGS in the presence of NaOH (at 850 ºC for 2 h) yields foamed materials with pores size larger than that of LGS-foam synthesized in the absence of NaOH. Although the addition of 1 wt.% CaCl_2_ increases the pore size, the addition of higher content (up to 5 wt. %) negatively affects the pore growth. Nevertheless, the use of AW in the presence of 3 wt.% CaCl_2_ enhances the foaming process.

Table 3 represents the common pores diameters and number of pores within foamed materials. For the samples synthesized at a temperature range of 750–850 ºC in the presence and absence of chemical additives, there is an inverse relationship between pores size and number of pores. The foamed sample with bigger pores size possesses the lower pores number, which is in line with previously published work (Xia et al. 2013). This rule is not applied at high temperature (950 ºC), as the low number of small sized pores have been identified. The melting of softened material could be the main reason behind this outcome.

**Table 3:** Common pore diameter and number of pores within foamed-LGS mixtures of 4 cm^2^ area

Mixture notation	Common pore diameter, mm	Number of pores
F-LGS-750 (2 h)	0.43	2775.00
F-LGS-800 (2 h)	0.75	391.00
F-LGS-850 (1 h)	1.02	315.00
F-LGS-850 (2 h)	1.75	118.00
F-LGS-850 (3 h)	1.94	98.00
F-LGS-950 (2 h)	0.36	69.00
F-LGS-850 (2 h)-SH1	2.31	99.00
F-LGS-850 (2 h)-SH3	3.03	59.00
F-LGS-850 (2 h)-SH5	4.15	20.00
F-LGS-850 (2 h)-CC1	2.37	58.00
F-LGS-850 (2 h)-CC3	1.04	118.00
F-LGS-850 (2 h)-CC5	0.62	760.00
F-LGS-850 (2 h)-CC3 / AW	4.28	20.00

**Fig. 4:**
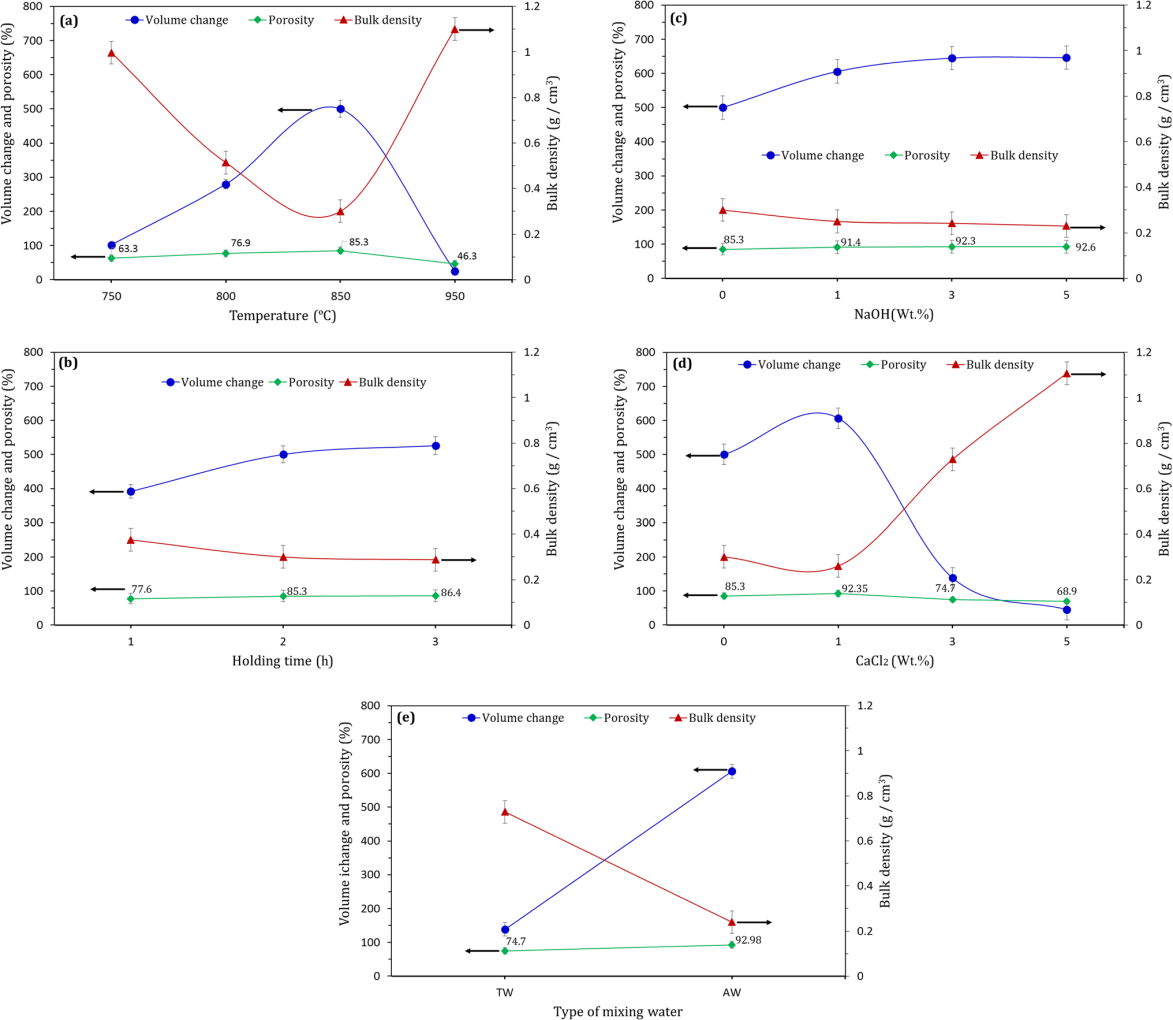
Relationship between volume change, porosity, and bulk density of foamed glass synthesized at (**a**) different sintering temperatures at 2 h holding time, (**b**) 850 ºC for different holding times, (**c** & **d**) 850 ºC and 2 h holding time in the presence of different NaOH and CaCl_2_ contents, respectively, and (**e**) 850 ºC and 2 h holding time in the presence of 3 wt.% CaCl_2_ individually dissolved in TW and AW before sintering process

It is undoubted that the emission of CO_2_ gas and the softening LGS during thermal treatment cause a remarkable increase in the volume of the sintered sample, resulting in a lightweight material with high porosity (Soloki and Esmailian 2015; Rajak et al. 2019; An et al. 2021). Accordingly, there is a strong correlation between volume change, porosity, and bulk density. The sample with the highest porosity exhibits the lowest bulk density and the highest volume expansion. As shown in Fig. 4a, increasing sintering temperature up to 850 ºC has resulted in a remarkable increment in the volume of the sample and porosity, accompanied by a significant reduction in bulk density. A further increase in sintering temperature leads to the formation of relatively dense sample with low porosity and high bulk density. Same trend was observed in the previous findings (Chen et al. 2017; Saparuddin et al. 2020). The change in holding time of at 850 ºC from 1 to 2 h yields thermally treated sample with higher volume expansion, higher porosity, and lower bulk density (Fig. 4b). The exposure of LGS to the same temperature up to 3 h causes a slight change in its properties, suggesting the fact that 850 ºC and 2 h are the optimum temperature and holding time, respectively, for foaming process.

Comparing with the foamed material synthesized at 850 ºC for 2 h holding time, the addition of 1 wt.% NaOH results in a considerable change in volume (500 to 605%), bulk density (0.3–0.25 g/cm^3^), and porosity (85.3–91.4%) (Fig. 4c). A slight change in these values has been recorded when the LGS sintered in the presence of higher NaOH contents (3 and 5 wt. %). Like NaOH with 1 wt.%, the use of CaCl_2_ with the same content also significantly improved the properties of the produced LGS-foam (Fig. 4d). A further increase in CaCl_2_ causes a negative effect on the foaming process, including the retardation of volume change, reduction of porosity, and increasing bulk density. Using AW as a mixing water to the sample having 3 wt.% CaCl_2_ was found to produce foaming materials with higher volume and porosity as well as lower bulk density (Fig. 4e).

**Fig. 5:**
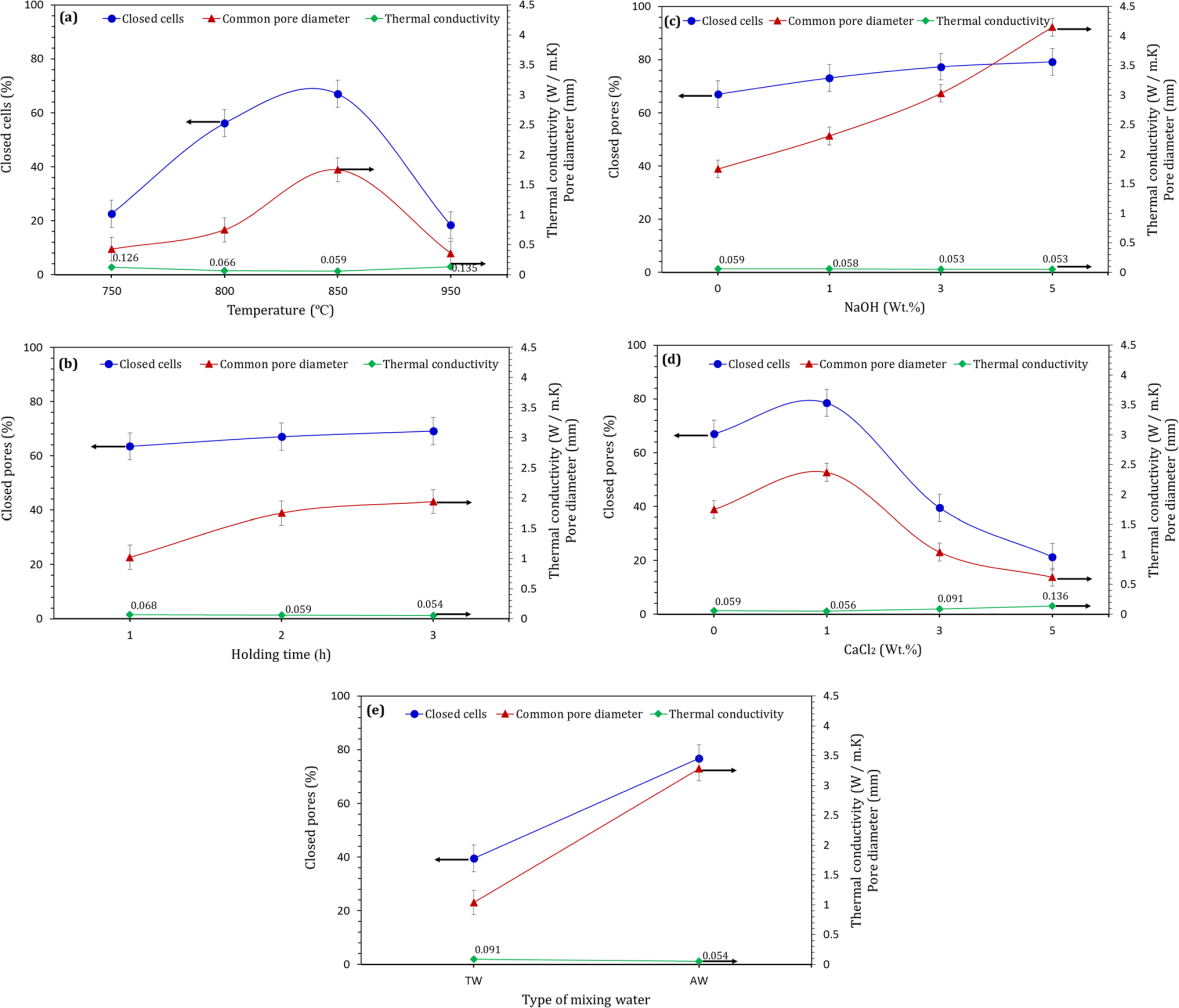
Relationship between thermal conductivity, common pore diameter, and closed cells percentage of foamed glass synthesized at (**a**) different sintering temperatures at 2 h holding time, (**b**) 850 ºC for different holding times, (**c** & **d**) 850 ºC and 2 h holding time in the presence of different NaOH and CaCl_2_ contents, respectively, and (**e**) 850 ºC and 2 h holding time in the presence of 3 wt.% CaCl_2_ individually dissolved in TW and AW before sintering process

Thermal conductivity is regarded as one of the main parameters which measure the capability of porous materials for insulating the heat (Ge and Zheng 2020; Assefi et al. 2021). It is well known that the thermal conductivity mainly depends on the porosity of foamed material, as the sample with highest porosity shows the highest thermal insulation (Haihui et al., 2017; Hassan et al. 2018; König et al. 2018). Herein, other parameters such as common pores diameter and closed cells percentage within foamed materials were represented in Fig. 5(a–e). A reverse relationship between thermal conductivity, closed cells percentage and common pores diameter has been detected. Increasing pores diameter and closed cells content materially decreased the thermal conductivity value. This can be explained by following facts (König et al. 2019): As the movements of air or CO_2_ gas entrapped by closed cells are restricted, the increased closed cells content inhibit heat transfer by convection through the cellular structure. Furthermore, the increase in pore diameter of foamed material with a homogeneous pore structure results in decreasing the pore walls solid volume and hence limiting the heat transfer by conduction and radiation.

Rising sintering temperature up to 850 ºC results in the increase of closed cells content and diameter of pores accompanied by thermal conductivity reduction. However, the exposure of LGS to 950 ºC causes an opposite effect in which a dense structure has been obtained (Fig. 5a). The holding time was found to have a significant effect on thermal conductivity reduction, common pores diameter and closed cells% increment (Fig. 5b). The increase of exposure time results in the dissociation of greater amount of CaCO3 and hence the release of more CO2 gas. Owing to the lower thermal conductivity of CO_2_ enclosed within cells/ pores than static air (Vesovic et al., 1992), it contributes to the reduced in thermal conductivity of foamed-LGS. The same trend was recorded with increasing NaOH addition (Fig. 5c). Interstingly, the addition of 1 wt.% CaCl_2_ causes the formation of foamed-LGS with lower thermal conductivity escorted by higher closed cells% and larger common pores diameter than those of foamed material without CaCl_2_ at the same sintering temperature and holding time (Fig. 5d). The continuation of CaCl_2_ addition yields foamed-LGS with higher thermal conductivity, lower pores diameter and lower closed cells percentage. Nevertheless, the use of AW as alternative to TW in LGS having 3 wt.% CaCl_2_ enhances the thermal insulation of the produced foamed material, which associated with the formation of closed cells with higher diameter (Fig. 5e).

### Pb-leaching from foamed materials

Apart from the variations in Pb-concentrations, all values are below the safe limit of characteristic leaching procedure (TCLP: ≤ 5 mg/l) (Table 4). The untreated sample represents Pb-concentration of 93 mg/l. The exposure of LGS to different elevated temperatures decreases the leachability of Pb. Specifically, the foamed materials sintered at 750, 800, 850, and 950 ºC demonstrate Pb-concentration of 3.75, 3.01, 2.12, and 1.78 mg/l with immobilization% of 95.98, 96.77, 97.72, and 98.09%, respectively. Increasing holding time from 1 to 3 h at the sintering temperature of 850 ºC decreases the Pb-concentration from 2.78 to 2.03 mg/l. A significant decrease in Pb-concentration was recorded when the foaming process takes place in the presence of NaOH and CaCl_2_. The foamed materials having CaCl_2_ showed the lowest Pb-concentrations. A slight reduction in Pb-leachability was got when CaCl_2_ dissolved in AW before sintering process.

**Table 4:** Pb-leaching behavior of LGS and the synthesized foamed LGS through sintering process at different conditions

Mixture notation	Pb-concentration, mg/l	Immobilization, %	Pb-fractions, %			
			Exchangeable	Bound to organic	Acid soluble	Non bioavailable
LGS	93.18	0.00	9.22	6.52	20.62	63.64
F-LGS-750 (2 h)	3.75	95.98	-	-	8.25	91.75
F-LGS-800 (2 h)	3.01	96.77	-	-	6.05	93.95
F-LGS-850 (1 h)	2.79	97.00	-	-	7.03	92.97
F-LGS-850 (2 h)	2.22	97.61	-	-	4.13	95.87
F-LGS-850 (3 h)	2.03	97.82	-	-	3.94	96.06
F-LGS-950 (2 h)	1.78	98.09	-	-	3.68	96.32
F-LGS-850 (2 h)-SH1	1.53	98.35	-	-	2.25	97.75
F-LGS-850 (2 h)-SH3	0.87	99.06	-	-	1.45	98.55
F-LGS-850 (2 h)-SH5	0.76	99.18	-	-	1.38	98.62
F-LGS-850 (2 h)-CC1	0.99	98.93	-	-	1.01	98.99
F-LGS-850 (2 h)-CC3	0.51	99.45	-	-	0.51	99.49
F-LGS-850 (2 h)-CC5	0.30	99.67	-	-	0.36	99.64
F-LGS-850 (2 h)-CC3 / AW	0.12	99.87	-	-	0.29	99.71

Table 4 also shows that the Pb-fractions (Pb-concentrations in different leachates) of the foamed-LGS immersed in different media. Sintering LGS at 750 ºC leads to the transformation of all exchangeable Pb-fraction and Pb bound to organic matter to non-bioavailable Pb. Additionally, a significant decrease in the concentration of acid soluble Pb was recorded. A continues decrease in acid soluble Pb-fraction accompanied by a remarkable increment in non-bioavailable Pb was observed by rising temperature and by elongating sintering time. Although the addition of NaOH induces the transformation of low stable acid-soluble fraction into non-bioavailable Pb-fraction with higher stability, the use of CaCl_2_ shows the highest effect at all addition levels. Using AW, as a mixing water before sintering process, causes a marginal change in both acid soluble and non-bioavailable Pb-fractions within sintered-LGS having 3 wt.% CaCl_2_, indicating that the type of water has low effectiveness in Pb-stabilization within sintered LGS-CaCl_2_ system. It is recommended that, the volatized Pb as PbCl_2_ should be collected in seal vessel containing CO2 to yield cerussite and hydrocerussite phases with higher stability..

**Fig. 6:**
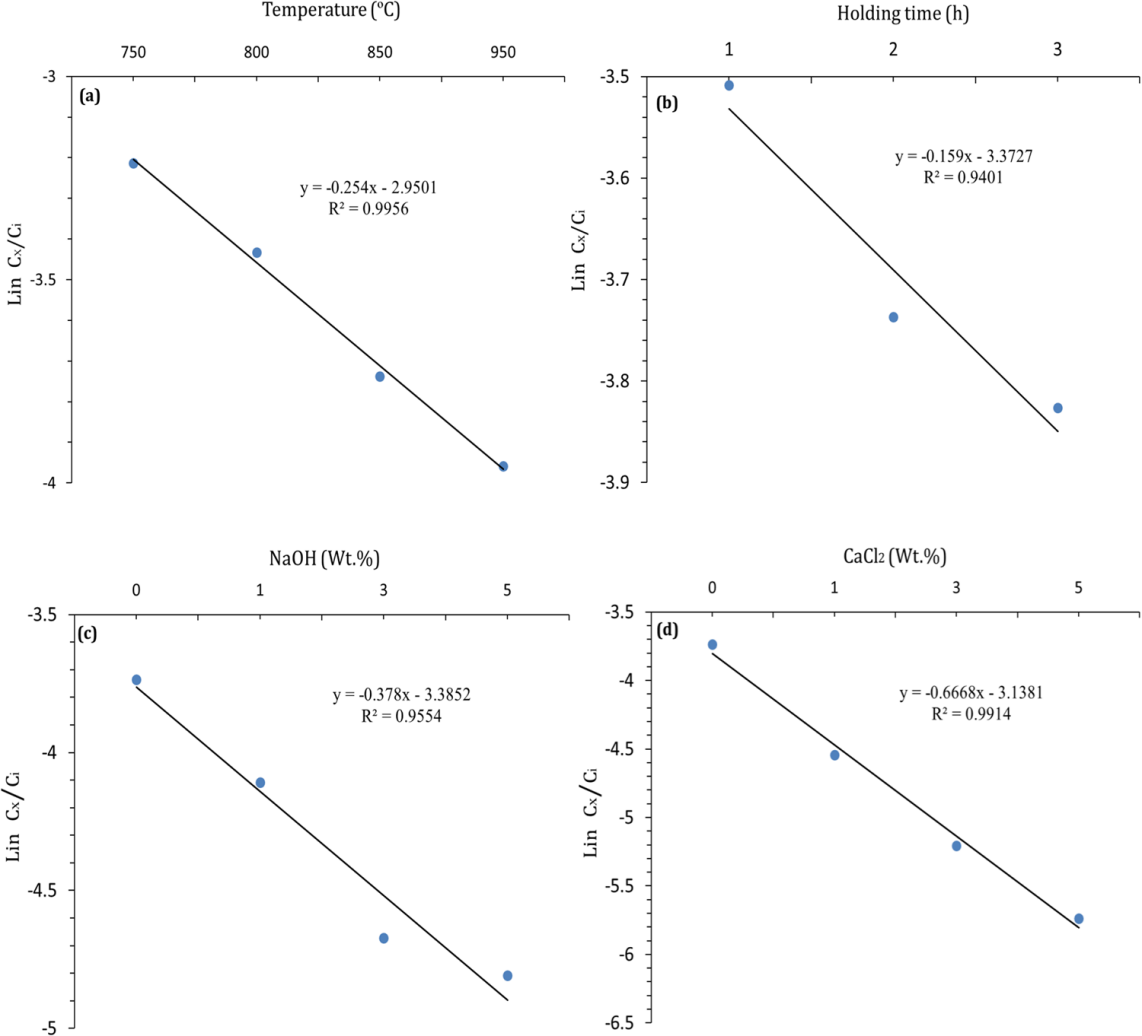
Lin Cx/Ci values as a function of (**a**) sintering temperatures, (**b**) holding times, and (**c** & **d**) NaOH and CaCl_2_ contents, respectively

First-order kinetic reaction was detected by plotting ln Cx/Ci (Ci and Cx are the Pb-concentrations before and after sintering process, respectively) as a function of temperature, holding time, and NaOH and CaCl_2_ contents (Fig. 6a–d). The simple statistical model shows that the effect of sintering temperature represents rate constant (K) value (0.254) higher than that of holding time (0.159). Incorporating NaOH was found to increase the *K*-value (0.378); however, CaCl_2_ has a higher effect (0.666). As a rule, *K*-value is beneficially used to identify the impact of the independence parameters on the Pb-stabilization and/or removal. The higher the *K*-value, the higher the effect of the independence parameter on the Pb-stabilization/-removal (Abdel-Gawwad et al. 2020b). The effectiveness of such parameters on the immobilization/removal process can be ordered as follows: CaCl_2_ > NaOH > sintering temperature > holding time.

## Discussion

**Fig. 7:**
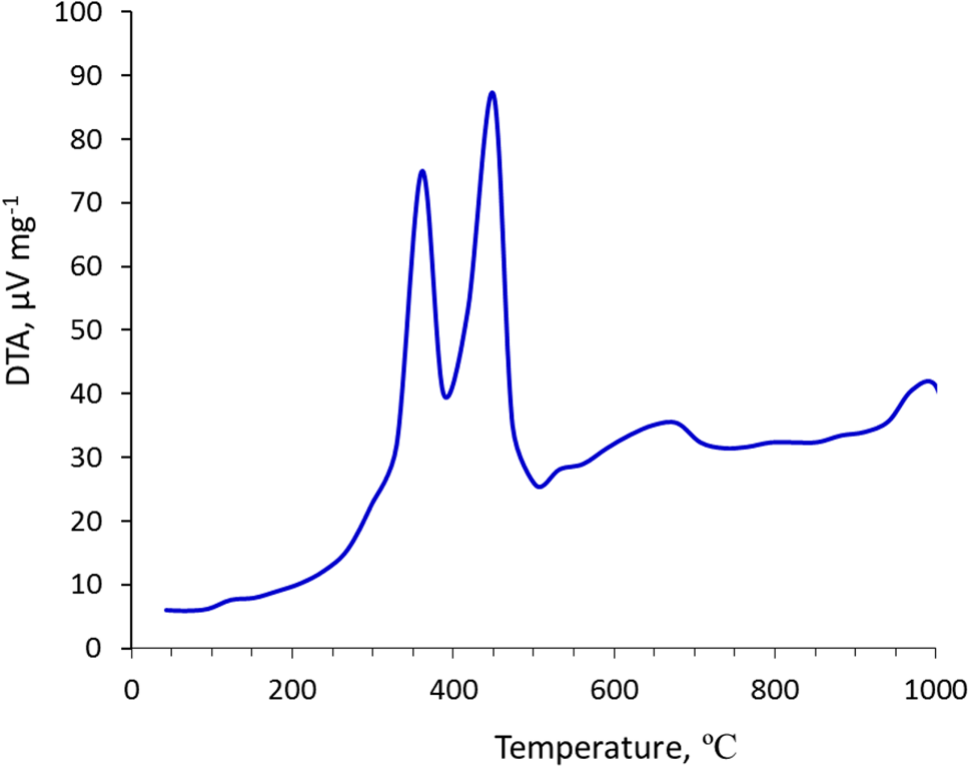
DT-curve of LGS

The transformation of hazardous LGS into safely used thermally insulating product via sintering/chlorination process is the main goal of this work. DT-curve (Fig. 7) shows that the organic matter within LGS completely dissociates at 500 ºC; meanwhile, CaCO_3_ decomposes at a temperature range of 700–840 ºC. The LGS was completely softened at 840 ºC. Therefore, the organic matter does not contribute to the foaming process, as the gas resulted from the decomposition of organic matter sublimates before LGS-softening. Accordingly, the foaming process strongly influenced by synergistic CaCO_3_-calcination and silicate- softening. The sintering temperature of 850 ºC offers the favorable conditions for supplementing the decarbonation and softening processes accompanied by the formation of foamed materials with high volume, high porosity, low bulk density and low thermal conductivity. Nevertheless, an opposite effect were detected when the sintering temperature increases up to 950 ºC, which could be explained by the transformation of softened material to molten one, yielding material with low-volume change, low porosity, high bulk density, and high thermal conductivity.

**Fig. 8:**
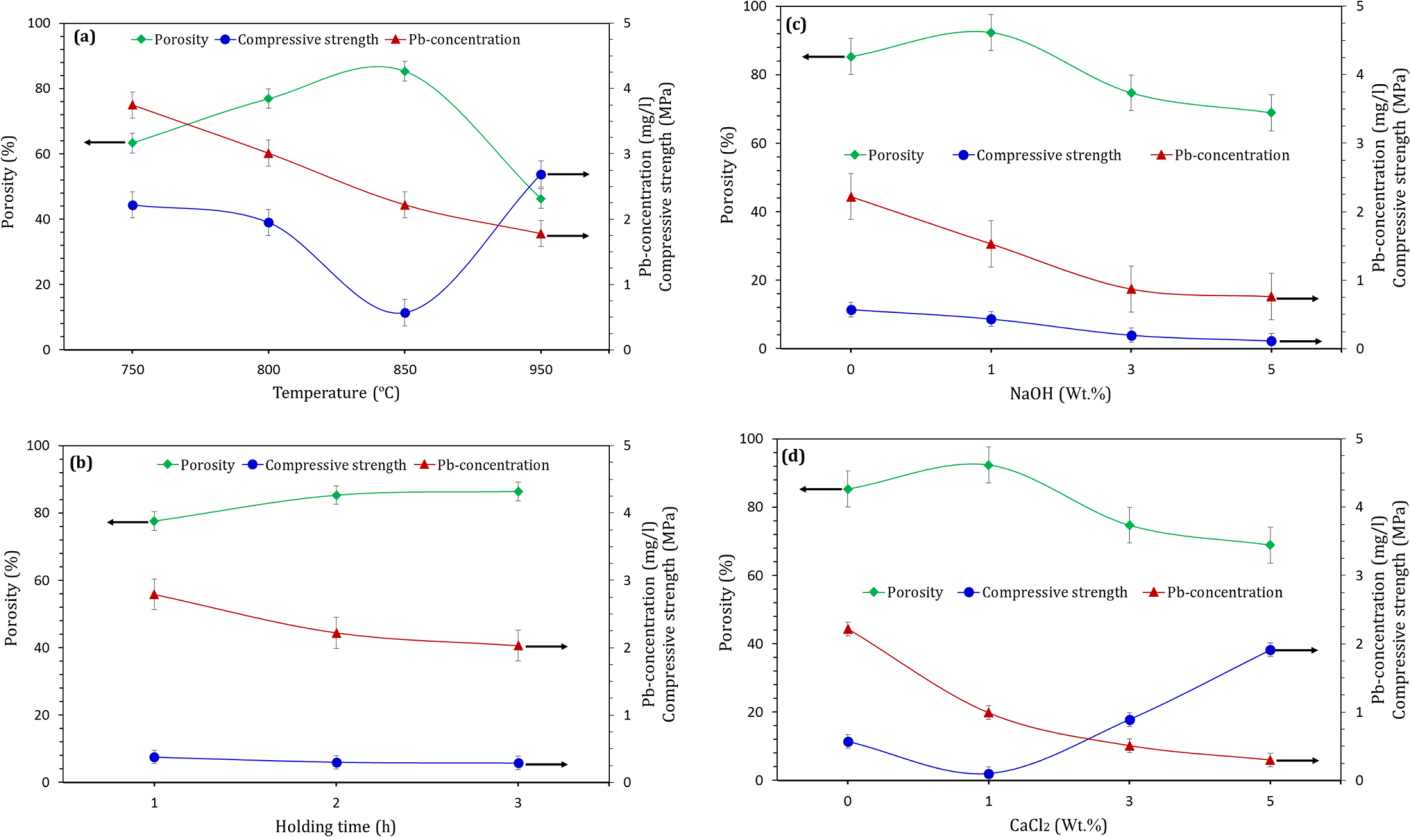
Relationship between Pb-concentration, compressive strength, and porosity of foamed glass synthesized at (**a**) different sintering temperatures, (**b**) different holding times, and (**c** & **d**) different NaOH and CaCl_2_ contents, respectively

The exposure of LGS to elevated temperature not only yields foamed materials with highly thermal insulating property but also strongly affects the reduction of free Pb within LGS. Several previously published works (Nikolić et al. 2014; Wang et al. 2020; Abdel-Gawwad et al. 2020c; Long et al. 2021) proved that the Pb-leaching strongly influenced by the compaction and porosity of the hardened materials. The hardened material with the highest compressive strength and lowest porosity exhibits the highest efficiency in Pb-immobilization. An opposite trend was detected in the present work, as Pb-leaching not influenced by the porosity and compressive strength of the produced foamed-materials (Fig. 8a–d).

**Fig. 9:**
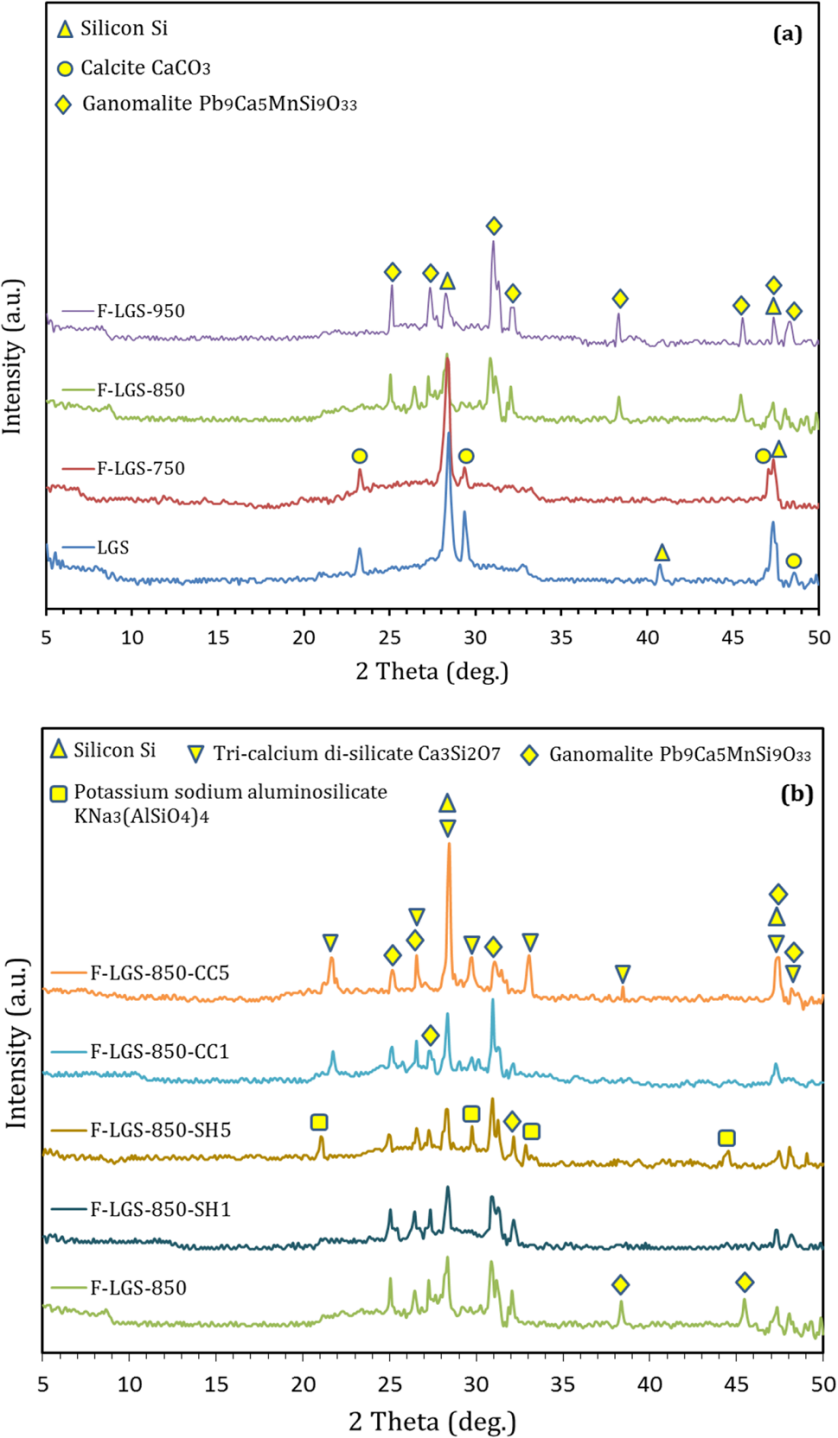
XRD-patterns of foamed materials fabricated at (**a**) different elevated temperatures and (**b**) in the presence different NaOH and CaCl_2_ contents

To shed more light on the reasonable reason behind the low Pb-leaching from highly porous foamed-LGS, the XRD-analysis was conducted on the sintered LGS at different conditions. Figure 9a shows that the intensity of CaCO_3_ decreases after the exposure of LGS to 750 ºC, indicating the partial decomposition of CaCO_3_. No calcium oxide (resulted from calcination of CaCO_3_) peaks have been detected but new reflections affiliated to ganomalite phase Pb_9_Ca_5_MnSi_9_O_33_ have been observed. This means that the CaO resulted from the calcination of CaCO_3_ consumed in the formation of crystalline ganomalite phase. Increasing temperature has resulted in the disappearance of CaCO_3_-peak, the reduction of silicon peak, and an enhancement in ganomalite peak intensity. Interstingly, the reduction of silicon could be caused by the oxidation reaction at elevated temperatures to yield silicon dioxide (Nguyen 2011; Gerlach and Maser 2016), which could contribute to the formation of ganomalite phase.

As stated by Nowak et al. (2012); Yu et al., (2013), Wang et al., (2017); Li et al., (2019), the use of chloride salts (NaCl and/or CaCl_2_) as an additive to Pb-contaminated-soil was found to be more effective in Pb-removal through chlorination and volatilization process. This explains the continuous reduction of Pb-leaching after the addition of CaCl_2_ to LGS before thermal treatment. Based on chlorination/volatilization process, the evaporated PbCl_2_ could contribute to the foaming process like CO_2_ resulted from CaCO_3_ calcination. These outcomes confirmed that the low leaching of Pb from sintered-LGS is caused by the positive effect of thermal treatment on Pb-stabilization (through the formation of ganomalite phase with high stability) and Pb-removal (through the chlorination/volatilization of Pb).

As confirmed by XRD-analysis (Fig. 9b), the addition of CaCl_2_ enhances the formation of tri-calcium di-silicate (Ca_3_Si_2_O_7_) phase at the expense of ganomalite one (responsible for Pb-stabilization). Nevertheless, increasing CaCl_2_ addition greatly decreased Pb-leaching. This should be explained by the decrease of free Pb-availability, which associated with the high efficiency of CaCl_2_ in the removal of Pb through the enhancement of chlorination/evaporation process. Although it enhances the Pb-removal, the high content of CaCl_2_ causes the retardation of foaming process, yielding high dense/low porous foamed materials. This ascribed to the formation of calcium silicate phase with higher sintering temperature (Zhou et al. 2017). Utilizing AW as a solvent for CaCl_2_ instead of TW was found to enhance the foaming process, suggesting the AW acts as fluxing material which decreases the sintering temperature of LGS-CaCl_2_ system.

Regardless of NaOH (as one of the common fluxing materials) enhances the softening and foaming process under thermal load, it also significantly decreases the Pb-concentrations in leachates. All sintered LGS synthesized in the presence of NaOH represent the same features, including reducing silicon peak with the formation of ganomalite phase (Fig. 9b). Nevertheless, a new phase affiliated to potassium sodium aluminosilicate was observed. NaOH was found to have high efficiency in the dissolution of amorphous silicate network, yielding reactive silicate species which easily interacts with free Pb to yield lead silicate with high stability, as in line with previously published works (Abdel-Gawwad et al. 2019). Although the effectiveness of NaOH in the enhancement of foaming process and in the immobilization of Pb, it exhibits high environmental pollution. Each tonne of NaOH generates approximately 1.91–3.18 tonnes CO_2_ (Turner and Collins 2013; McLellan et al. 2011). The utilization of AW, as an alternative to NaOH, not only decreases the CO_2_ emission but also reduced the processing cost of the fabricated foams.

The inferior thermal conductivity coefficients of the foamed-LGS composites point out their superior thermal resistivity. All the composites sintered at 800 and 850 ºC with and without additives (NaOH or CaCl_2_) possessed thermal conductivity values ≤ 0.1 W/m.K (prescribed limit of thermal insulation); however, the composite sintered at 850 ºC for 3 h exhibited the lowest thermal conductivity (0.054 W/m.K); i.e., generally they can be classified as good thermal insulators and recommended for various applications in the thermal insulation of buildings. The significantly reduced thermal conductivity with the reasonable compressive strength and adequate thermal stability could point out the enhanced durability; these findings could explain the competitive advantages of the developed novel composites over the organic foam materials like (polystyrene, polyurethane, etc.) which lack of sufficient mechanical strength and suffer from low melting temperature.

## Conclusions

In this study, the successful synthesis of well-defined and homogeneous porous foamed glass with minimum to non-detectable levels of Pb-leaching has been attained using a rapid and facile method. Lead glass sludge is composed of amorphous lead silicate materials with considerable calcium carbonate content, which acts as interior foaming agent. several findings can be concluded as follows:

1. The exposure of lead glass sludge to elevated temperature (sintering) has resulted in the formation of porous foamed material with low Pb-leachability. Additionally, the immobilization process was induced by the interaction of free lead and amorphous silicate within lead glass sludge with calcium oxide (resulted from calcium carbonate calcination) to yield insoluble ganomalite mineral with high stability as confirmed by X-ray diffraction.
2. The simple kinetic model proved that calcium chloride addition showed the highest efficiency in the reduction of Pb-leaching via synergistic stabilization (by the formation insoluble ganomalite) and chlorination/vaporization (by the formation of PbCl_2_) process. Nevertheless, adding high calcium chloride content has a negative impact on foaming process which associated with the formation of calcium silicate mineral.
3. Increasing sodium hydroxide addition enhanced the foaming process and Pb-immobilization. Alkali wastewater has been successfully used as an alternative to sodium hydroxide because it enhanced the foaming process of lead glass sludge system containing 3wt. % calcium chloride. Tuning the pore size and structure of the foamed glass is doable via varying sintering conditions and foaming agent content.
4. Thermal conductivity of the produced foamed glass mainly influenced by the type of pores (closed or open), pore size, and pore distribution. The thermal conductivity values are within the prescribed limits for thermal insulation materials.
5. The experimental results did not reveal a correlation between the porosity of the synthesized foamed glass and Pb-leachability, suggesting the fact that the transformation of exchangeable Pb into insoluble Pb-containing-phase is the dominant immobilization mechanism.
6. The obtained results indicate that the newly developed foamed glass composites are of great importance and may be adopted for thermal insulation in several applications.
7. Although the proposed method showed the high efficiency in the immobilization/ removal Pb, it represents a main shortcoming as the sublimated PbCl_2_ through sintering process causes an environmental problem. It recommended to design an innovative system to control in the vaporized toxic gas.

## Data Availability

All data and materials will be available upon request.
